# The impact of the field width on VMAT plan quality and the assessment of half field method

**DOI:** 10.1002/acm2.12834

**Published:** 2020-02-20

**Authors:** Berat Tugrul Ugurlu, Oztun Temelli

**Affiliations:** ^1^ Department of Radiation Oncology Faculty of Medicine Inonu University Malatya Turkey

**Keywords:** VMAT, field width, half field, virtual phantom, nasopharyngeal carcinoma

## Abstract

**Purpose:**

The goal of this work is to investigate the field width dependence of the volumetric modulated arc therapy (VMAT) plan quality and to propose a half field method to irradiate large volumes effectively with VMAT.

**Materials and methods:**

We compared four different VMAT methods; namely three full field (3ff), four full field (4ff), three half field (3hf), four half field (4hf). To evaluate the impact of the field width on VMAT plan quality, 12 different size PTVs were created in the virtual phantom and treatment plans generated for each PTV were compared. The effectiveness of our half field method was tested using computed tomography (CT) data of 10 nasopharyngeal carcinoma patients.

**Results:**

In the virtual phantom study, organs at risk (OAR) mean dose, the maximum point dose, and Homogeneity Index (HI) were found to be field width dependent. Conformation Number (CN) was not significantly affected. In the clinical study, 4hf plans obtained statistically significant dose reduction at brainstem (*P* < 0.001), right parotid (*P* = 0.034), oral cavity (*P* < 0.001), larynx (*P* = 0.003), cochlea (*P* = 0.017), lips (*P* = 0.024), and Body‐PTV (*P* = 0.04) compared to 4ff plans.

**Conclusion:**

Our results indicate that VMAT plan quality is dependent on the field width. Half field VMAT method, with the help of reduced field width, shows a clear advantage for the irradiation of large size targets compared to traditionally used full field VMAT plans.

## 1 Introduction

A novel form of radiotherapy called intensity modulated arc therapy (IMAT) was proposed by Yu in 1995^1^ and its clinically applicable implementation, called as volumetric modulated arc therapy (VMAT), was developed by Otto in 2008.^2^ Since then, it has been received broad interest mostly due to its reduced monitor units (MU) and treatment delivery time compared to intensity modulated radiation therapy (IMRT).^3^, ^4^, ^5^, ^6^, ^7^ Otto's VMAT technique relies on a continuous modification of the gantry speed, the dose rate and the multileaf collimator (MLC) position to modulate the delivered radiation.^2^ Initially, a coarse sampling method was used to optimize dynamic gantry motion. However, Otto proposed a progressive sampling method to optimize gantry and MLC positions. According to this method, rotational delivery of radiation is modeled by as a series of static fields. At the beginning of the optimization, a small number of evenly distributed static field samples is initialized. After some iterations, an additional set of field samples is added to the optimization process. Field samples are continuously added until the whole gantry span is successfully covered.^2^

In theory, VMAT can deliver a fraction dose in a single rotation. However, for complex shaped target volumes more than one rotation is required to achieve equivalent results compared to IMRT.^8^ This is mainly caused by constraints using by the optimization engine. Efficiency constraints, like MLC leaf positions and MU weights,^2^ preserve continuous and fast delivery and restrict the optimization engine.

**Figure 1:**
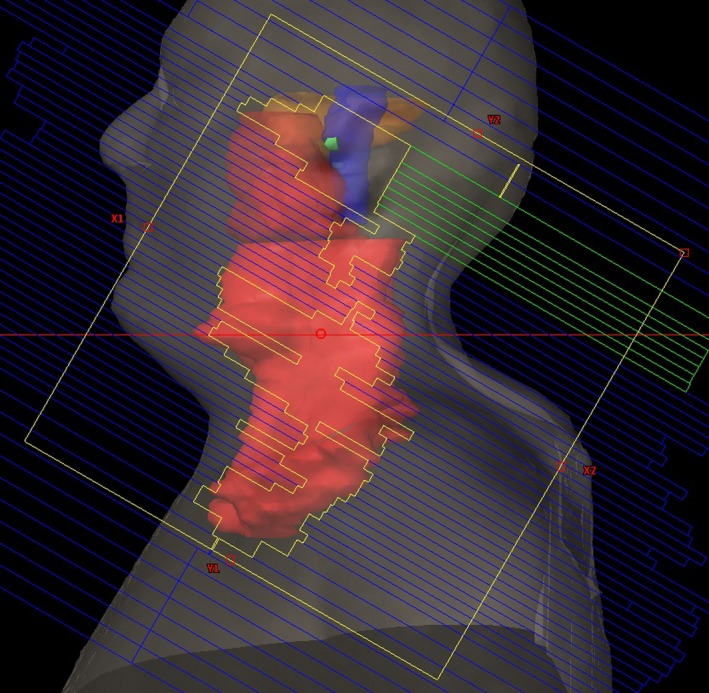
A BEV at gantry angle 92°. PTVs are shown as red contour. The selected MLC leaves (highlighted as green) reached their maximum traveling distance, thus cannot cover brain stem (blue contour), temporal lobe (yellow contour), and cochlea (green contour). The field width was 25 cm

Beside MLC leaf positions and MU weights, there are several more restrictions in VMAT optimization. The physical limitations of the machine, such as the dose rate variation and the maximum leaf speed, are directly taken into account in the optimization process. However, there is at least one more limitation: the MLC leaf length. It especially gains importance in large VMAT fields. Varian Millennium MLC (Varian Medical Systems, Palo Alto, CA, USA) leaves are 15 cm long (at isocenter), so two opposite MLC leaves, moving parallel to the x‐jaws, can only cover up to 30 cm of the field. Thus, MLC leaf length directly limits x‐jaw aperture of VMAT fields. Because of this limitation, Varian Eclipse^TM^ Treatment Planning System (TPS), may offer a multi‐isocentric treatment plan, which requires multiple setups. Multiple setups should be avoided whenever possible, since they may increase the treatment time and potential for set‐up errors.^9^ Additional to this, when a VMAT field has an x‐jaw aperture more than 15 cm, then one leaf cannot cover the whole field by itself. In other words, it cannot reach the opposite end of the field. This sometimes causes undesirable irradiation of the healthy tissue and degrades the plan quality. One example can be seen from beam's eye view (BEV) of a nasopharyngeal carcinoma VMAT plan (see Fig. 1).

The physical limitations of the treatment machine and the efficiency constraints of VMAT restraint freedom required to get higher quality treatment plans. The purpose of this study is to investigate the impact of the field width (x‐jaw aperture) on VMAT plan quality and to propose a mono‐isocentric treatment method for irradiation of large targets with VMAT. This method is referred as half field VMAT.

## 2 Materials and Methods

### 2.1 Treatment planning strategies

We used a similar approach that has been investigated with flattening filter free beams for breast cancer to create half fields.^10^ We define the following methods: three full field (3ff), four full field (4ff), three half field (3hf), four half field (4hf). The number in the names of the plans refers to the number of rotations used. In full field plans, jaw apertures are selected so that planned target volume is fully covered at all gantry angles. Half field plans are generated from full field plans by blocking half of the fields. To get half blocked fields, one x‐jaw is closed at the center of the field. The same x‐jaw was blocked whenever possible to avoid high and low dose regions at the junctions. The strategy underlines these four different treatment techniques is as follows: 3ff is considered to be a baseline treatment plan that is widely accepted in clinical applications. 4ff benefits from one more rotation that provides extra freedom to get more desirable dose distribution. 3hf uses two half blocked fields and one full field (Since half fields require complementary field to cover the whole target, the third field must be fully opened). Half blocked fields have reduced field widths; therefore, MLC leaves travel shorter distance and reach higher modulation capability. 4hf benefits from the advantages of four half blocked fields.

Plans were created on the Eclipse^TM^, version 13.7.14 (Eclipse, Varian Medical Systems, Palo Alto, CA, USA). All plans were calculated using the Anisotropic Analytical Algorithm (AAA) version 13.7.14. The dose calculation grid was set to 1.25 mm. All plans were generated by using 6 MV photon beams from a Varian Trilogy iX linear accelerator equipped with a 120 leaf Millennium MLC which has a leaf length of 15 cm at isocenter.

### 2.2 Virtual phantom case

**Figure 2:**
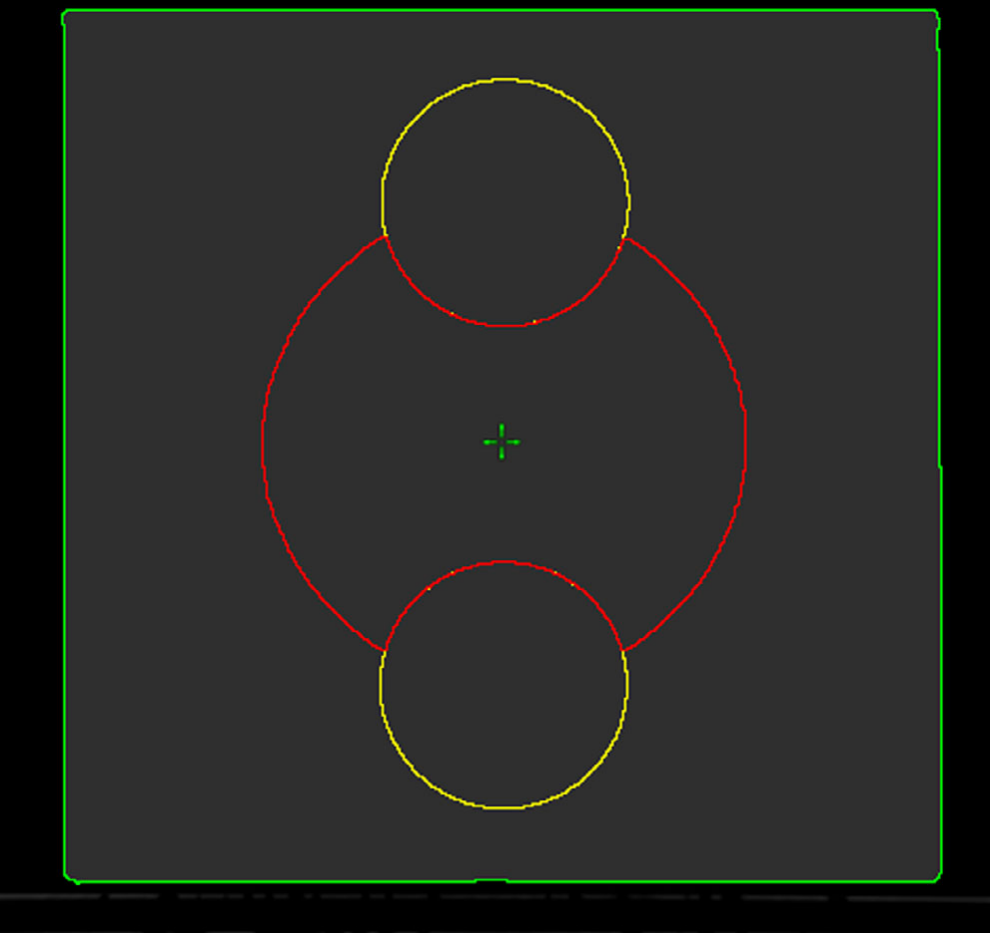
Axial view of the virtual phantom. 25 cm spherical PTV (red) and its corresponding OAR (yellow) contour can be seen

A homogeneous water equivalent virtual phantom (had the width and height of 45 cm) was created within the Eclipse TPS. To evaluate the impact of the field width on VMAT plan quality, 12 spherical planning target volumes (PTVs) and corresponding spherical organs at risk (OARs) were contoured (see Fig. 2). Each PTV had a different diameter (7, 10, 13, 16, 19, 22, 25, 28, 31, 34, 37, 40 cm) and located at the center of the virtual phantom. For each PTV, two OAR contours (will be regarded as a single parallel organ both in the optimization and the dose calculation process) were created at the anterior and posterior sides of the PTV and located so that their center is on the border of the PTV contour. Then each PTV contour cropped from its corresponding OAR to crate convex shaped PTV structure. The diameter of each OAR contour set equal to the half of their corresponding PTV's diameter. Corresponding OARs for PTV 34, 37, and 40 cm did not completely fit into the virtual phantom. Hence, exterior parts of them were cropped which reduced their volumes and gave the optimization an additional difficulty.

Four techniques (3ff, 4ff, 3hf, and 4hf) described above were applied to 12 PTVs. In total 48 VMAT treatment plans were generated. The prescription dose was 30 Gy in 10 fractions. All plans were normalized so that 95% of the PTV gets the prescribed dose. The dose volume constraints and priorities used in the optimizations were kept the same and are included in Table 1.

**Table 1:** Optimization parameters used in the virtual phantom optimization

Structure	Dose objective	Priority
PTV	D_99.9_> 30 Gy	200
	D_0.1_ < 32.5 Gy	200
OAR	Mean dose < 15 Gy	150
Virtual phantom	D_0_ < 33 Gy	500

Dx, the dose to x% of the structure.

### 2.3 Clinical case

To test the effectiveness of half field method in a clinical case, a retrospective analysis was performed on computed tomography (CT) data of 10 nasopharyngeal carcinoma patients. CT data sets were acquired with a 3 mm slice thickness. Patients were in supine position and their heads and shoulders immobilized by head and neck immobilization masks.

Three target volumes were defined according to the Radiation Therapy Oncology Group 0225 protocol.^11^ The gross tumor volume (GTV) was defined as all known gross disease including primary cancer and nodes. The clinical target volumes (CTVs) was defined as the GTV plus areas considered to contain potential microscopic disease. The CTV_70_ (for the delivery of an absorbed dose of 70 Gy) includes the GTV and 5 mm isotropic margin. The CTV_59.4_ was defined as 5mm margin around the CTV_70_ plus areas at risk, including the entire nasopharynx, retropharyngeal lymph nodal regions, clivus, skull base, pterygoid fossae, parapharyngeal space, inferior sphenoid sinus, and posterior third of the nasal cavity and maxillary sinuses. The CTV_54_ includes clinically negative low‐neck regions. 5 mm margin applied to define the PTV_70_ and the PTV_59.4_. The PTV_54_ was equal to the CTV_54_. For the regions where the GTV was adjacent to brain stem, 1 mm margin was applied for the generation of the CTV. The dose prescription was 70 Gy to the PTV_70_, 59.4 Gy to the PTV_59.4_, and 54 Gy to the PTV_54_, in 33 fractions delivered using a simultaneous integrated boost technique. All plans were normalized so that 95% of the PTV_70_ gets the prescribed dose.

Since 4ff plans expected to give more desirable dose distribution, the 4ff plan was first optimized. ALARA (as low as reasonably achievable) principle was used in the optimization process. The optimization objectives were adjusted until reaching a trade‐off plan. After a successful 4ff plan was obtained, other plans were optimized by using the same optimization criteria to ensure a fair comparison between different techniques. All plans were generated and optimized by the same physicist.

### 2.4 Data analysis and Comparison Criteria

All plans were quantitatively evaluated using dose–volume histogram (DVH) analysis. In dose comparison, we used the maximum point dose, homogeneity index (HI), Van't Riet's conformation number (CN),^12^ mean and maximum point doses of OARs, mean dose of the body and total monitor unit (MU). HI was defined as (D_2_ − D_98_)/D_50_ where D_x_ is the dose received by x% of the target volume. CN was calculated as (V_T,ref_/ V_T_) × (V_T,ref_/ V_ref_), where V_T_ is the target volume, V_T_,_ref_ is the volume of the target receiving at least the reference dose, and V_ref_ is the total volume receiving at least the reference dose.

In the virtual phantom case, we only considered HI, CN, organ mean dose, and the maximum point dose as comparison criteria. To visualize the quality of the plans we used a plan quality index (QI):

$$\text{QI}\;=\sum_{\text{criterion}}{\text{IF}}_{\text{criterion}}-\left({\text{Value}}_{\text{particular}}-{\text{Value}}_{\text{best}}\right),$$

where IF_criterion_ is the importance factor of a criterion (HI, CN, organ mean dose and the maximum point dose), Value_particular_ is the value of that criterion obtained by particular treatment technique, Value_best_ is the best value of that criterion achieved by any one of four techniques (3ff, 4ff, 3hf, and 4hf). Importance factors are determined by a radiation oncologist and were 20 for HI, CN, and the maximum point dose of PTV and 40 for the organ mean dose. Higher QI values indicate higher quality VMAT plans in terms of factors considered.

In the clinical case, for each PTV (PTV_70_, PTV_59.4_ and PTV_54_), HI was calculated by the same formula given above. CN was only calculated for the PTV_70_. Brain stem, cord, optic nerves, chiasm, cochlea, parotid glands, oral cavity, larynx, eyes, lenses, temporal lobes, and lips were considered as OARs.

The statistical analyses were performed using IBM SPSS statistics for Windows version 22.0. Shapiro‐Wilk test was used to test the normality. For normally distributed data the Paired Samples T‐test and for non‐normally distributed data the Wilcoxon Signed Ranks Test were performed. Statistical significance level was set to *P* < 0.05.

## 3 Results

### 3.1 Virtual phantom case

**Figure 3:**
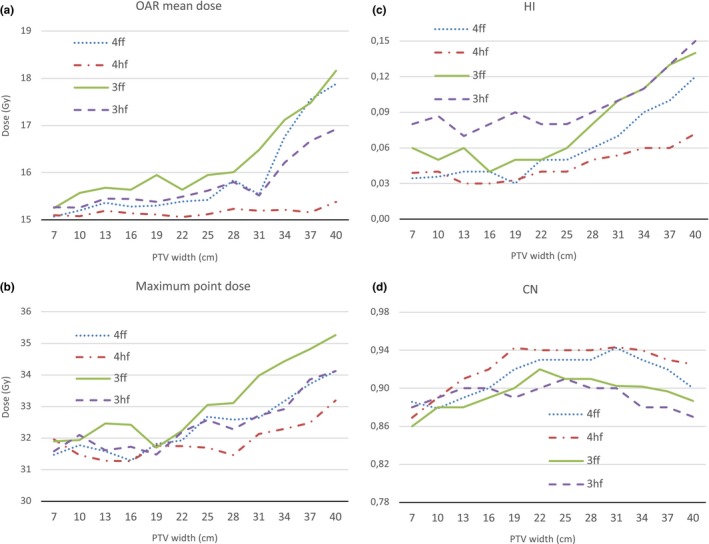
Comparison of four different methods in terms of (a) OAR mean dose, (b) the maximum point dose, (c) HI and (d) CN

**Figure 4:**
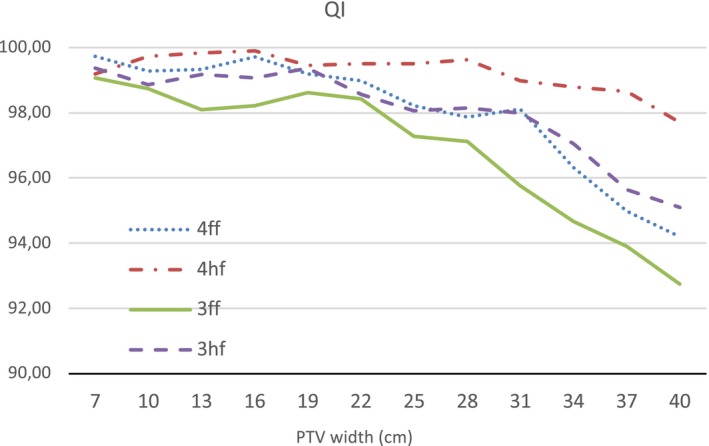
QI values of four different methods

The smallest PTV has a diameter of 7 cm and a volume of 93.7 cm^3^, whereas the largest PTV has a diameter of 40 cm and a volume of 30124.1 cm^3^. Fig. 3 presents OAR mean dose, the maximum point dose, HI and CN for 3ff, 4ff, 3hf, and 4hf plans. Fig. 4 provides QI values.

OAR mean dose increases quickly for all plans of PTV larger than 33 cm except 4hf method. Reduced OAR mean dose for all PTVs were achieved by 4hf method (see Fig. 3). The maximum point dose increases as PTV size increases, and it was found to be lower in 4hf plans. The planning aim that maximum point dose should be less than 33 Gy was met by 4hf method for all PTVs. 3ff plans showed highest maximum point doses for all PTVs. Similarly, 4hf plans obtained better HI values. On the other hand, there was no significant difference in terms of CN and all techniques were able to preserve an acceptable value for CN even for large size PTVs. 3ff technique provided clinically acceptable plans until the PTV width reaches 24 cm. After that, the plan quality deteriorates quickly to unacceptable levels as it can be seen from QI values. 4ff method gives somewhat better dose distribution than 3ff. However, for PTVs, larger than 24 cm, the decrease in plan quality can be seen in 4ff plans as well. In half field plans QI values were higher than full field plans in average and 4hf gave higher QI value for PTVs larger than 7 cm. Additionally, the best values for OAR mean dose, the maximum point dose, HI and CN were obtained in 4hf plans. The highest QI value was obtained in 4hf plan for 16 cm PTV.

MU values and the virtual phantom mean doses were presented in Table 2. Half field plans used significantly more MU, while virtual phantom mean doses were almost the same in all methods.

**Table 2:** MU values and virtual phantom (VP) mean doses

Method	MU	VP mean dose (Gy)
3ff	921	11.8
3hf	1453	11.7
4ff	1137	11.8
4hf	2305	11.6

### 3.2 Clinical case

**Figure 5:**
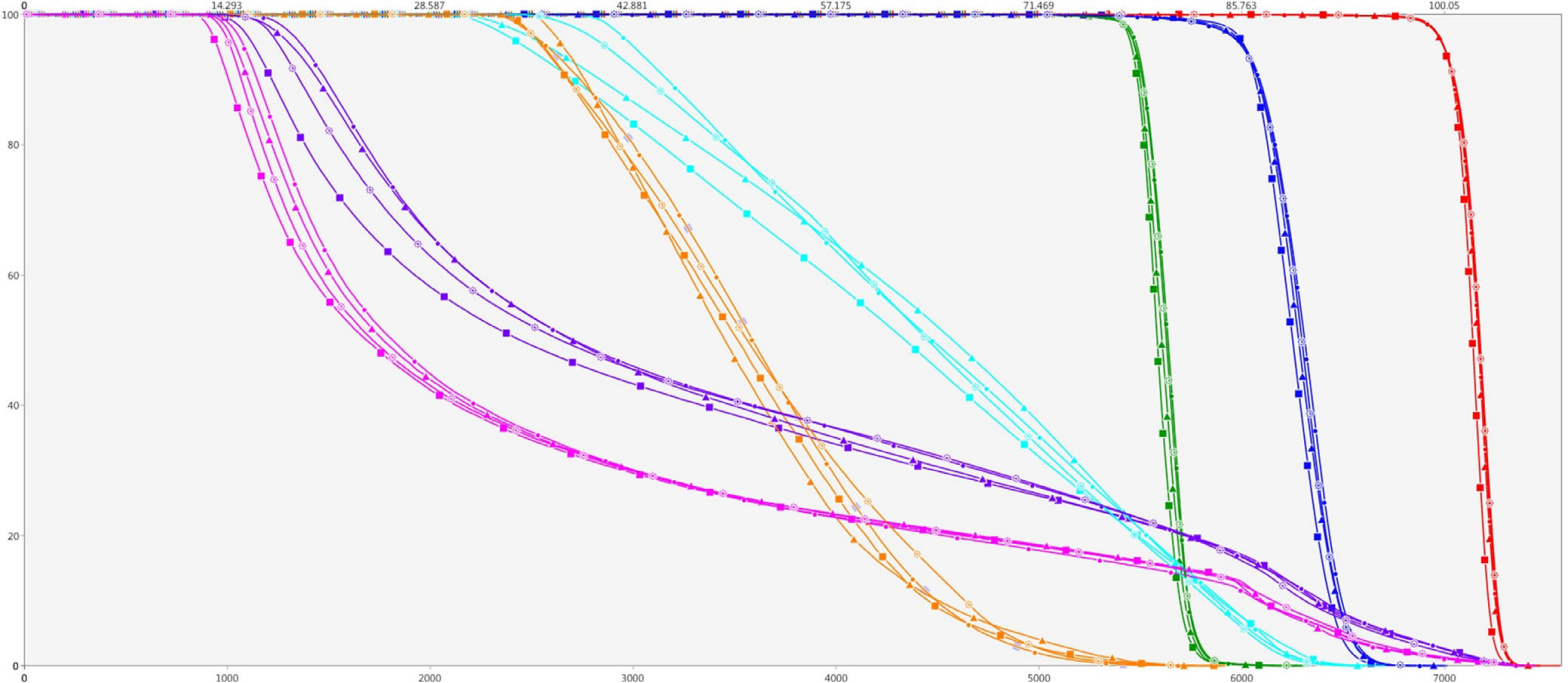
A typical DVH comparing 3ff (star), 3hf (triangle), 4ff (circle) and 4hf (square) plans for PTV70 (red), PTV59.4 (blue), PTV54 (green), oral cavity (cyan), larynx (yellow), left parotid (purple), right parotid (meganta)

Within the studied patients; the mean volumes of the PTV_54_, PTV_59.4_, and PTV_70_ were 207 ± 203 cm^3^, 412 ± 143 cm^3^, and 85 ± 40 cm^3^ respectively. Average x‐jaw apertures were 21.4 cm, 21.2 cm, 14.0 cm, and 10.7 cm for 3ff, 4ff, 3hf, and 4hf plans respectively. Average y‐jaw apertures were almost same being 22.3 cm, 22.0 cm, 21.5 cm, and 21.3 cm for 3ff, 4ff, 3hf, and 4hf plans respectively. Fig. 5 shows comparative dose–volume histogram (DVH) of one patient. Within four different techniques, 4hf showed a clear advantage in terms of homogeneous dose coverage as indicated by the sharp downfall of the PTVs curve. Table 3 indicates the detailed statistical analysis on PTVs. 4hf reached better HI in comparison of 4ff (*P* = 0.035) and it reached significantly lower mean dose of the PTV_70_ (*P* = 0.021 compared to 4ff), PTV_59.4_ (*P* < 0.001 compared to 4ff), and PTV_54_ (*P* = 0.039 compared to 4ff). 3hf method achieved significantly lower PTV_54_ mean dose compared to 3ff. There was no statistically significant difference in other criteria.

**Table 3:** Statistical analysis on PTV coverage

Structure	3ff	3hf	Sig (p) (3ff ‐ 3hf)	4ff	4hf	Sig (p) (4ff ‐ 4hf)
PTV_70_						
mean dose (Gy)	71.43 ± 0.23	71.52 ± 0.23	0.304	71.57 ± 0.25	71.16 ± 0.16	0.021
HI	0.05 ± 0.01	0.05 ± 0.01	0.417	0.05 ± 0.01	0.04 ± 0.01	0.441
CN^a^	0.90 (0.83‐0.91)	0.90 (0.88‐0.92)	0.188	0.90 (0.88‐0.91)	0.90 (0.88‐0.91)	0.807
PTV_59.4_						
mean dose (Gy)	62.72 ± 0.27	62.73 ± 0.2	0.105	62.92 ± 0.24	62.46 ± 0.2	<0.001
HI^a^	0.12 (0.08‐0.15)	0.10 (0.08‐0.14)	0.385	0.10 (0.08‐0.13)	0.09 (0.07‐0.12)	0.035
PTV_54_						
mean dose (Gy)	56.18 ± 0.29	55.94 ± 0.18	0.031	56.1 ± 0.2	55.92 ± 0.17	0.039
HI^a^	0.06 (0.05‐0.09)	0.06 (0.05‐0.07)	0.287	0.07 (0.06‐0.07)	0.06 (0.05‐0.09)	0.834

a Wilcoxon signed Ranks Test was used for non‐normally distributed data, otherwise Paired Samples T‐test was used.  Underlined values indicates statistical significance (*P*<0.05).

Table 4 shows results of statistical comparisons on OAR doses. High sparing of OARs near the targets was achieved by half field plans. There was a slight dose reduction in the maximum point dose at brain stem and cord with 3hf (0.3 Gy and 0.4 Gy median dose reduction at brain stem and cord, respectively) and 4hf (0.5 Gy and 0.4 Gy median dose reduction at brain stem and cord, respectively) plans. Although the maximum dose of cord was found to be lower for half field plans, the difference was not significant (*P* > 0.05). Equivalent findings were obtained for the maximum point dose at chiasm, optic nerve, lens, cochlea, and temporal lobe maximum doses (*P* > 0.05 for all comparisons). Half field plans achieved slightly reduced maximum dose at eye, while 3hf plans showed significant dose reduction compared to 3ff (*P* = 0.022).

**Table 4:** Statistical analysis on OAR doses

Structure	3ff	3hf	Sig (p) (3ff ‐ 3hf)	4ff	4hf	Sig (p) (4ff ‐ 4hf)
Maximum dose (Gy)						
Brain stem^a^	52.2 (51.6‐53.9)	51.9 (51.1‐53.5)	0.053	51.8 (50.9‐53.9)	51.3 (50.4‐52.3)	<0.001
Cord^a^	43.4 (42.0‐45.4)	43.0 (42.3‐44.4)	0.188	42.9 (42.0‐45.4)	42.5 (42.1‐43.6)	0.138
Chiasm^a^	48.7 (11.8‐53.2)	50.5 (18.4‐51.5)	0.868	48.6 (13.2‐51.8)	50.0 (22.4‐51.3)	0.918
Optic nerve^a^	43.5 (21.9‐50.4)	44.5 (29.3‐50.8)	0.884	42.3 (27.9‐50.7)	45.4 (28.2‐50.4)	0.767
Cochlea^a^	46.3 (43.6‐61.1)	45.5 (44.2‐57.0)	0.246	45.6 (43.8‐60.0)	44.9 (36.7‐56.4)	0.131
Lens	6.6 ± 1.4	6.5 ± 1.1	0.188	6.6 ± 1.2	6.4 ± 1.2	0.063
Eye	31.4 ± 7.2	29.6 ± 7.8	0.022	30.5 ± 7.0	28.4 ± 6.9	0.108
Temporal lobe^a^	63.6 (49.6‐70.3)	63.8 (50.7‐69.4)	0.648	62.6 (49.6‐68.9)	64.0 (49.2‐68.6)	0.839
Mean Dose (Gy)						
Left parotid	27.8 ± 6.9	27.4 ± 7.3	0.053	27.6 ± 6.9	26.8 ± 7.0	0.005
Right parotid	25.1 ± 3.1	24.4 ± 3.0	0.045	24.6 ± 2.9	24.0 ± 2.7	0.034
Oral cavity^a^	41.2 (38.4‐51.0)	41.4 (38.2‐51.0)	0.024	40.7 (38.1‐49.4)	39.8 (38.0‐49.4)	<0.001
Larynx^a^	30.6 (27.2‐37.5)	31.2 (28.2‐36.1)	0.562	32.5 (27.5‐37.2)	29.3 (25.2‐37.1)	0.003
Cochlea^a^	38.7 (35.9‐55.4)	37.4 (33.9‐48.4)	0.038	37.3 (34.4‐53.8)	35.4 (32.1‐50.8)	0.017
Eye	8.5 ± 1.8	8.1 ± 1.7	0.116	8.0 ± 1.5	8.2 ± 1.8	0.616
Lips^a^	20.2 (14.8‐40.1)	18.6 (13.4‐34.2)	< 0.001	19.1 (13.3‐37.7)	16.7 (12.3‐32.4)	0.024
Body‐PTV	20.2 ± 1.4	19.8 ± 1.5	0.031	20.2 ± 1.6	19.7 ± 1.4	0.04

a Wilcoxon signed Ranks Test was used for non‐normally distributed data, otherwise Paired Samples T‐test was used. Underlined values indicates statistical significance (*P* > 0.05).

Half field plans obtained almost 1 Gy dose reduction for the mean doses of parotid glands. 4hf plans showed the lowest mean doses at left and right parotids (26.8 and 24.0 Gy respectively). The same observation was found for the mean doses of oral cavity and larynx. For oral cavity, the mean dose was reduced 0.9 Gy with 4hf technique compared to 4ff (*P* < 0.001) and for larynx, the mean dose reduction was 3.7 Gy with 4hf technique (*P* = 0.003). The mean dose of the cochlea was also reduced significantly for both half field plans (*P* = 0.038 for 3hf in comparison of 3ff; *P* = 0.017 for 4hf in comparison of 4ff). The observed difference on mean dose of eye were not statistically significant. For the mean dose of lips, half field plans showed a significant difference and 4hf method achieved the lowest mean dose at lips being 16.7 Gy (*P* = 0.024).

The mean dose of healthy tissue was found to be higher in full field plans. Similar to other results, 4hf obtained the lowest mean dose of healthy tissue being 19.7 Gy (*P* = 0.04). The dose reduction was 0.5 Gy with 4hf plans compared to 4ff plans and 0.4 Gy with 3hf plans compared to 3ff plans. 3ff and 4ff plans used 474 and 503 MU while 3hf and 4hf plans used 690 and 818 MU in average.

## 4 Discussion

We have first analyzed the impact of the field width on VMAT plan quality. Comparing the plans generated for different size PTVs in the virtual phantom, traditional three field VMAT plans could only offer clinically acceptable plans for up to 25 cm large targets. For targets larger than that, the plan quality decreases quickly as it can be seen in QI values (see Fig. 4). 4ff method on average achieved better plan quality as compared with 3ff method. One extra rotation helped the optimization to get a more desirable plan. However, a similar degree of decrease in plan quality as the targets get larger was observed for 4ff plans too. OAR mean dose and the maximum point dose were highly affected from the field width. CN was the only comparison criteria that did not seem to be depended on the field width. For the smallest PTV, CN was found to be the lowest. This might be due to the challenge to reach high conformity in small convex shaped targets.

To irradiate large targets with VMAT, we have reported a mono‐isocentric half field VMAT method. Compared with traditionally used full field VMAT plans,^6^, ^13^, ^14^, ^15^, ^16^ our approach offers a potential to provide greater flexibility in the dose delivery. This is mainly due to the additional dose modulation capability in the fields having smaller x‐jaw apertures. Our half field method uses half blocked fields to reduce the field width. By shortening the traveling distances for MLC leaves, higher modulation and more desirable dose distribution are aimed.

Comparing our method with traditional full field plans generated for the virtual phantom, our method demonstrated higher robustness for target size. Still being dependent on the target size, both 3hf and 4hf plans had better QI overall. In the plans generated for 40 cm PTV, 4hf achieved 97.7 for QI while it was only 94.2 in 4ff plan. This difference demonstrates the capability of our half field method for large targets. The main contributors to this difference were OAR mean dose, the maximum point dose and HI. The 4hf method was able to maintain an acceptable level of OAR mean dose even for the plan generated for the PTV has 37 cm width. 3hf and 4hf plans obtained similar maximum point doses outperformed full field plans. 4hf achieved better HI for PTVs larger than 13 cm in comparison of full field plans. On contrary, half field method offered no improvement for CN. The 4ff method offered higher CN compared to 3hf and 4hf for PTVs larger than 22 cm.

We have tested our method in a clinical case. Comparing plans generated for nasopharyngeal carcinoma tumors, our method offers similar PTV coverage while reducing mean OAR doses. Using multiple rotations in VMAT has been reported to achieve superior target coverage compared with single arc VMAT.^8^ The findings were consistent. In overall four field plans generated better HI and lower target mean dose compared to three field plans. However, our half field plans were able to achieve even lower target mean dose. By using four half blocked fields, 4hf method was able to get the lowest HI. Comparing our half field method with other studies, HI was similar to that reported by Szu‐Huai et al^15^ for high dose PTV and better to that reported by Johnston et al^6^ for all PTVs. We achieved superior CN values comparing with previously reported studies.^13^, ^14^ However, all of our plans obtained clinically acceptable CN and we did not observe any statistically significant difference. For OAR doses, half field method achieved significant dose reductions especially for OARs located closer to the targets. 3hf and 4hf plans were able to reach statistically significant dose reduction at the mean doses of right parotid, oral cavity, larynx, cochlea, and lips. Chiasm, optic nerves, eyes, and lenses did not differ substantially with different methods. This might be due to the larger distance of these OARs to the targets.

The main drawback of our half field method is the increased MU. It uses higher MU than a typical three field VMAT plan and less MU than a typical 7‐9 field IMRT plan.^16^ MU is a measurement of the amount of radiation produced by the linear accelerator. Using higher MU results in increased scatter, which increases the dose to healthy tissue and potentially increases the risk of secondary cancers.^17^ However, irradiating large targets with VMAT requires the use of large fields. As it can be seen in Fig. 1, MLC leaves cannot cover large fields sufficiently. Because of the physical limitations of currently available treatment machines, using large fields in VMAT may cause undesirable direct irradiation of healthy tissue and OARs. Our findings indicate that, even though half field plans used considerably more MU, they have shown statistically significant dose reduction at the mean dose of healthy tissue and OARs.

As it can be seen from the QI values of the virtual phantom study, traditional full field method cannot offer clinically acceptable plans for large targets while it gives sufficient results for small targets. However, large targets are a part of modern radiotherapy. Bilateral breast irradiation, pelvic irradiation for gynecological malignancies, late stage prostate, scalp irradiation, and whole body irradiation require large fields. Our nasopharyngeal carcinoma study demonstrated that half field method has a potential to offer mono‐isocentric and effective treatment plans with VMAT technique.

This study only covers only Varian accelerators. Our half field method may not be applicable to other vendor's treatment machines. Jaw‐tracking was not available in our clinic at the time this study was conducted, and therefore it was excluded.

## 5 Conclusions

We have demonstrated the field width effect on VMAT plan quality and the capability of our half field method. It offers an improved dosimetric plan quality along with higher amount of MU usage. We also aimed to increase the usability of mono‐isocentric VMAT technique for large sized targets.

## CONFLICT OF INTEREST

No conflicts of interest.
